# Home Self-Massage Device Necessitates Public Awareness: Vertebral Artery Dissection Associated With a Home Massage Device

**DOI:** 10.7759/cureus.33394

**Published:** 2023-01-05

**Authors:** Erum Shariff, Ziyad T Al Ghannam, Fahad A AlDamigh, Abdulhadi G AlGhamdi, Yazan M AlEisawi, Khalid F Aloqalaa, Basil Z Sallout

**Affiliations:** 1 Neurology, Imam Abdulrahman Bin Faisal University, Dammam, SAU; 2 College of Medicine, Imam Abdulrahman Bin Faisal University, Dammam, SAU; 3 Medicine and Surgery, Imam Abdulrahman Bin Faisal University, Dammam, SAU

**Keywords:** stroke, posterior circulation stroke, massage, arterial dissection, vertebral artery (va)

## Abstract

Vertebral artery dissection (VAD) is a common cause of stroke in middle-aged individuals. Patients with VAD usually describe a trivial minor neck trauma preceding the event. Such traumas may be associated with spinal manipulation or sudden movements of the neck. Our case is a 43-year-old lady who presented with a history of sudden-onset dizziness, dysarthria, nausea/vomiting, tinnitus, and imbalance. Two days prior to her presentation, she experienced a new-onset moderate to severe intensity headache along with neck pain. The patient mentioned a first-time use of a home massage device three weeks prior to headache onset. After investigations, the patient was diagnosed with VAD, and treatment was initiated. She was discharged in stable condition. With the recent increased popularity of home massage devices, we report this case to raise awareness about the safe use of massage devices in order to prevent the occurrence of such injuries and complications.

## Introduction

Vertebral artery dissection (VAD) is considered to be an uncommon cause of stroke with an incidence of 2% of all ischemic strokes [1]. However, in middle-aged individuals (30-45 years of age), the incidence can be as high as 10% to 25% and is usually preceded by a minor or trivial trauma [1]. Other factors that may contribute to the development of VAD include genetic factors such as connective tissue disease and environmental factors that are associated with hyperextension or rotation of the neck, which includes yoga, ceiling painters, as well as factors associated with sudden neck movement sneezing and coughing. Furthermore, the clinical presentation of symptomatic VAD may include dizziness/vertigo, headache, gait problems, and other neurological symptoms related to posterior circulation [2].

In recent years, home massage devices have increased in popularity with the aim of relieving tightness or pain [3]. This further highlights the need to highlight the dangers of misusing such devices and encourage safe usage.

## Case presentation

**Figure 1:**
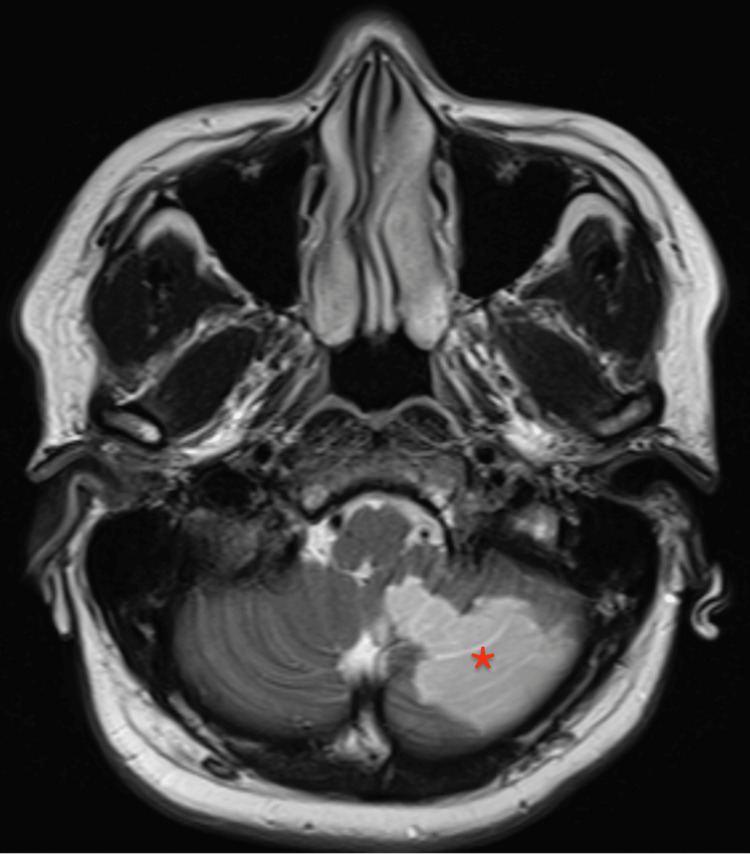
High T2 signal intensity involving the inferior part of the left cerebellum Asterisk: infarcted portion of the left cerebellum

A 43-year-old, right-handed lady with no known comorbid conditions presented to the emergency room (ER) with a two-hour history of sudden-onset dizziness, nausea/vomiting, tinnitus, and imbalance. She had a 30-minute history of left-sided hemiparesis that has resolved completely. For the previous two days, she experienced a new-onset moderate to severe intensity headache along with neck pain. The headache was unilateral in the occipital area. There was no history of trauma prior to this presentation but she mentioned a first-time use of a home massage machine about three weeks prior to symptoms onset. She worked as a physician and did not have a history of smoking, alcohol consumption, or any other chronic medical illnesses. Also, the patient did not have any family history of artery dissections, stroke at a young age, or inherited diseases (e.g. Marfan’s syndrome). On examination, the patient's vital signs were stable. She was a young female lying in bed comfortably. She was conscious, alert, and oriented to time, place, and person. Extraocular movements were intact with no nystagmus. She had equally reactive pupils, 2-3 mm bilaterally. Her facial sensation was intact; there was no facial asymmetry, centralized uvula, normal shoulder shrugging and sternocleidomastoid power, and no tongue deviation or atrophy. Her tone and power were normal bilaterally, with +3 deep tendon reflexes and a right-sided positive Babinski sign. Her coordination, gait, tandem gait, and Romberg tests were normal. Sensory examination was unremarkable. The initial diagnostic evaluation demonstrated a normal complete blood count, coagulation profile, and renal and liver function tests, apart from a mildly decreased hemoglobin level. Computed tomography (CT) head with CT perfusion (CTP) and CT angiogram (CTA) was performed. She had left cerebellar infarction in the posterior inferior cerebellar artery (PICA) territory. CTA demonstrated an abrupt cutoff of the contrast at the origin of the left vertebral artery (Figure 1), with faint contrast filling and irregular lumen seen within the V3 and V4 segments of the left vertebral artery, suggestive of VAD.

**Figure 2:**
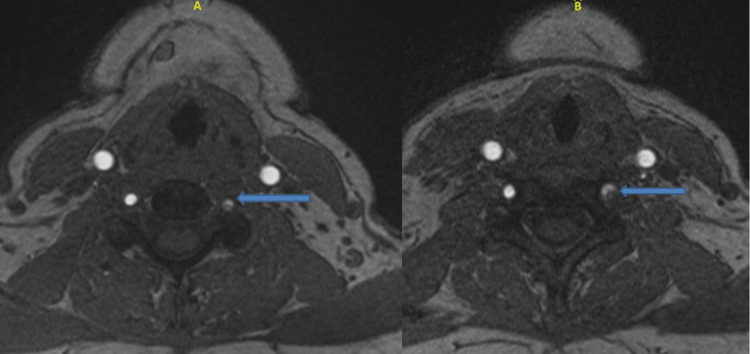
Crescent-shaped high T2 signal intensity within segment 4 of the left vertebral artery Blue arrows: the dissected part of the fourth segment of the left vertebral artery (LVA); picture A: upper cross-section magnetic resonance imaging (MRI) with contrast of the vertebral artery; picture B: lower cross-sectional MRI with contrast of the LVA

**Figure 3:**
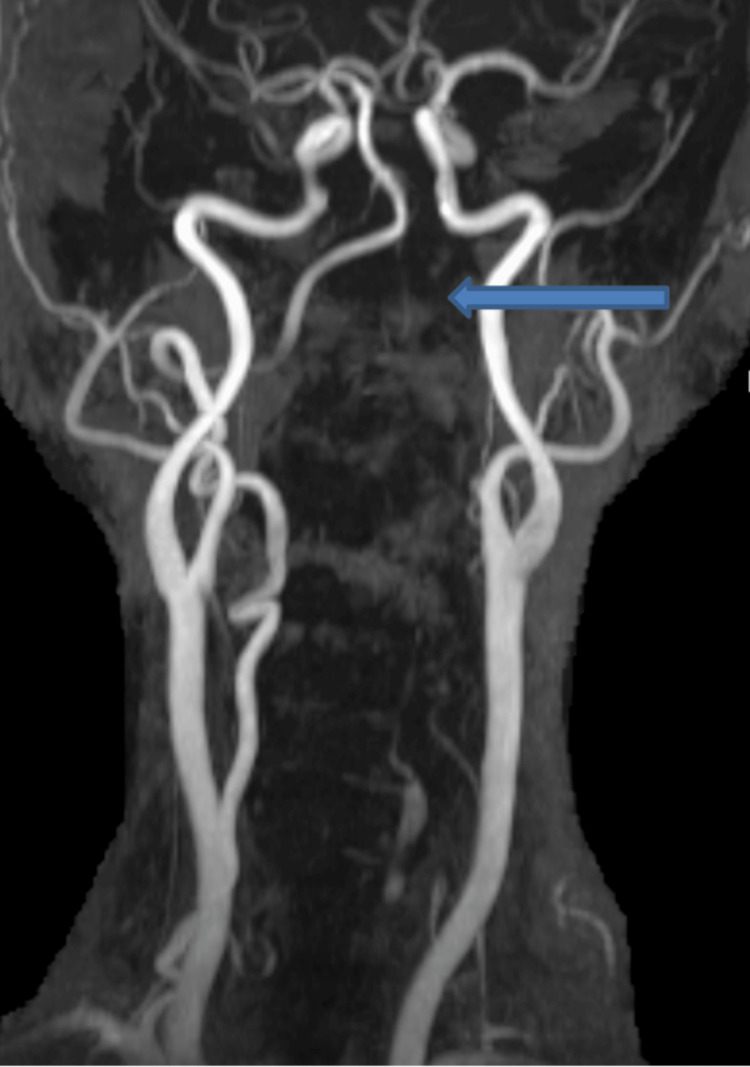
MRA showing significantly reduced flow within the left vertebral artery Blue arrow: reduced blood flow of the left vertebral artery (LVA)

Her National Institute of Health Stroke Scale (NIHSS) score was 0 upon arrival; however, on CTP she had a large penumbra in the territory of PICA. She was within the therapeutic window for intravenous tissue plasminogen activator (IV tPA) so she has been given IV tPA with no complications. She was admitted to the intensive care unit for an initial 24 hours observation. She had remained stable throughout her course in the hospital. Her repeat head CT did not reveal any hemorrhagic transformation. After 24 hours of IV tPA, she was started on acetylsalicylic acid 100 mg. The rest of her stroke workup was unremarkable, including the echocardiogram and Holter monitoring for 24 hours. Magnetic resonance imaging (MRI) and magnetic resonance angiogram (MRA) were both performed after three days and showed high T2 signal intensity involving the inferior part of the left cerebellum (Figure 1) and crescent-shaped T2 signal intensity in the fourth segment of the left vertebral artery (Figure 2). MRA demonstrated significantly decreased flow within the left vertebral artery (Figure 3). She was discharged from the hospital in stable condition.

**Figure 4:**
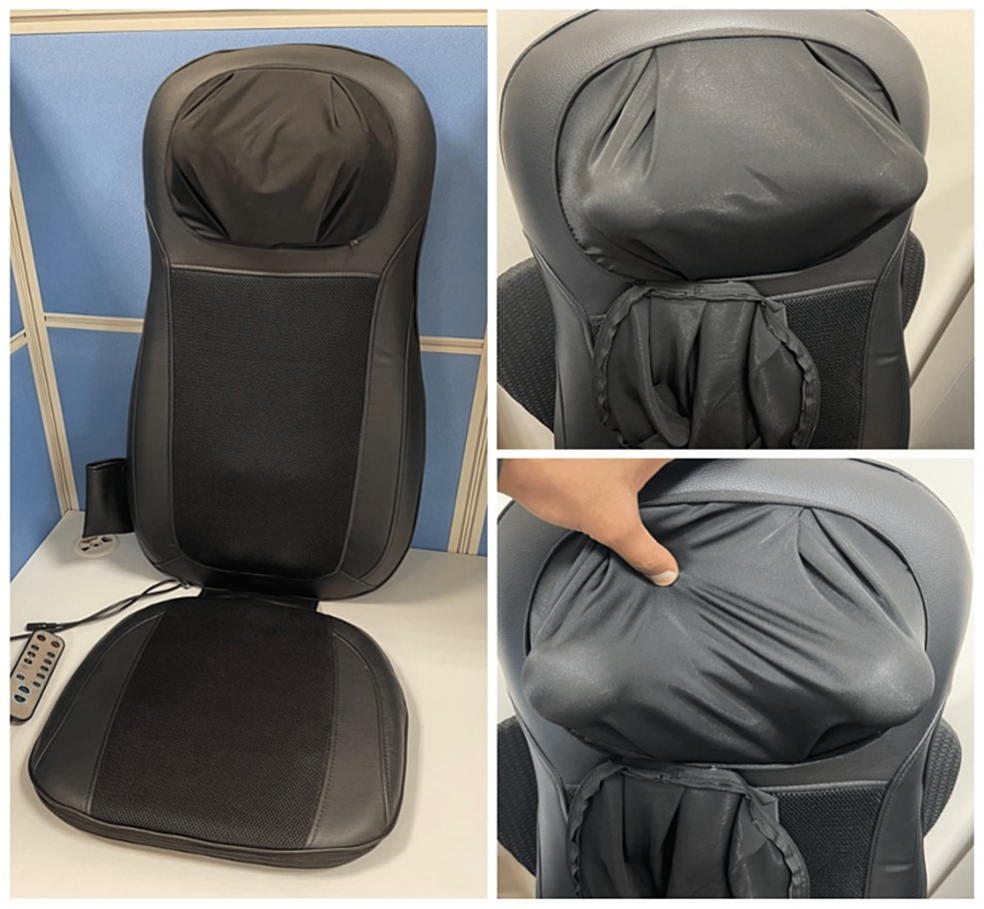
Home massage device with rotating probes in the neck region

The massage machine used was a “massage chair” with rotating probes to massage the back and neck (Figure 4). The instruction manual listed health conditions in which the device should not be used, including menstruation, pregnancy, heart disease, hemorrhagic disease, sepsis, and fever. The patient denied any of the previously mentioned conditions at the time of the use of the device. No information regarding safe use around the neck region or potential health risks was mentioned in the instruction manual.

## Discussion

It is great to gift yourself with a massage session after a long and tough working day, but what are the odds of having a cerebrovascular accident during a neck massage? VAD is a common cause of stroke in the young population with trauma, with strokes occurring in approximately 68% of patients with VAD [1,2]. However, VAD after massage sessions is not as common as other types of injury mechanisms like whiplash injuries. Even though, there are few case reports published lately about such mechanisms of injury [4].

VAD happens when the structural integrity of the arterial wall is compromised dissection happens. Intimal tears lead to arterial blood dissecting between the layers of the arterial wall. The blood within the arterial wall leads to hematoma and clot formation. The subsequent compromise in vertebral artery blood flow secondary to the stenosis leads to the symptoms of VAD [1].

Dissection and occlusion of the vertebral arteries can manifest in several neurological presentations, which range from mild to debilitating symptoms, including dizziness/vertigo, headache, neck pain, and gait abnormalities. The treating physician should have a high index suspicion because patients with VAD can present with a range of symptoms and and physical findings [2].

There is an increased popularity of the use of home-massage equipment in recent years with the aim of relieving tightness or pain [3,5]. Moreover, there is a wide range of handheld massage devices nowadays, and every single one has its unique massaging techniques and price. In a systematic review done across six countries, it was found that around 5.5% of adults visited a massage therapist as a form of complementary and alternative medicine in one year [6].

Our patient is a young female with no vascular risk factor, no history of trauma or neck surgery, and no history of smoking or alcohol use. The patient only gave a history of multiple recent usages of a massage device for the neck during the last two months,

VAD secondary to neck manipulation has been reported numerous times in literature. In a case report, a 39-year-old man presented with a gradual headache associated with nausea, vomiting, and neck pain, which started two days prior to his presentation after massage therapy. Investigations revealed bilateral dissection of the cervical segment of vertebral arteries with complete occlusion of the left vertebral artery and infarction in the left cerebellar hemisphere. The patient was managed with medical therapy and intensive physiotherapy and was discharged after four days [7]. In another case, a 30-year-old male patient suffered from headache, nausea, vomiting, blurred vision, diplopia, dizziness, and ataxia following an episode of a neck massage. Brain CT and MRI revealed acute infarction of the left cerebellar hemisphere. Digital subtraction angiography showed narrowing and dilatation of the V3 segment of the left vertebral artery with narrowing of the V4 segment consistent with dissection, along with a cavernous segment aneurysm of the contralateral internal carotid artery [8]. Moreover, in another case, a 35-year-old Chinese man with no risk factors for stroke presented to the ER complaining of right-sided body weakness for one day following expressive dysphasia for two days. The presentation was preceded by multiple sessions of neck, shoulder girdle, and upper back massage for pain relief in the prior two weeks. Investigations revealed left internal carotid artery dissection [9]. In another case report, a 27-year-old female presented with two weeks of progressively worsening dizziness in the form of vertigo and disequilibrium associated with neck pain and headache. The patient denied trauma but only gave a history of recent use of a handheld massage device on her neck recurrently over the past three weeks. Her investigation revealed vertebral artery dissection extending from the second to fifth cervical vertebras. The patient was admitted and managed with medical therapy. He was discharged after one day of being symptom-free. The patient did not show up for a follow-up [10].

Therefore, careful history-taking regarding low-energy traumas sustained from such equipment is warranted if VAD is suspected, as this would increase the suspicion of VAD and further investigations should be obtained to confirm the pathology. Furthermore, awareness regarding safe massage practices needs to be addressed as well as providing detailed instructions on how to use automated massage machines in order to prevent such potential complications.

## Conclusions

Careful history-taking regarding low-energy traumas sustained from home massage equipment is warranted if VAD is suspected, as this would increase the suspicion of VAD and further investigations should be obtained to confirm the pathology. Furthermore, awareness regarding safe massage practices needs to be addressed as well as the provision of a detailed instructions manual with safety precautions regarding the use of automated massage machines in order to prevent such potential complications.
